# Genome Scale Mutational Analysis of Geobacter sulfurreducens Reveals Distinct Molecular Mechanisms for Respiration and Sensing of Poised Electrodes versus Fe(III) Oxides

**DOI:** 10.1128/JB.00340-17

**Published:** 2017-09-05

**Authors:** Chi Ho Chan, Caleb E. Levar, Fernanda Jiménez-Otero, Daniel R. Bond

**Affiliations:** a BioTechnology Institute, University of Minnesota—Twin Cities, St. Paul, Minnesota, USA; b Department of Plant and Microbial Biology, University of Minnesota—Twin Cities, St. Paul, Minnesota, USA; c Department of Biochemistry, Molecular Biology, and Biophysics, University of Minnesota—Twin Cities, St. Paul, Minnesota, USA; Geisel School of Medicine at Dartmouth

**Keywords:** Geobacter, extracellular electron transfer, Tn-Seq, multiheme cytochrome, extracellular respiration

## Abstract

Geobacter sulfurreducens generates electrical current by coupling intracellular oxidation of organic acids to the reduction of proteins on the cell surface that are able to interface with electrodes. This ability is attributed to the bacterium's capacity to respire other extracellular electron acceptors that require contact, such as insoluble metal oxides. To directly investigate the genetic basis of electrode-based respiration, we constructed Geobacter sulfurreducens transposon-insertion sequencing (Tn-Seq) libraries for growth, with soluble fumarate or an electrode as the electron acceptor. Libraries with >33,000 unique insertions and an average of 9 insertions/kb allowed an assessment of each gene's fitness in a single experiment. Mutations in 1,214 different genomic features impaired growth with fumarate, and the significance of 270 genes unresolved by annotation due to the presence of one or more functional homologs was determined. Tn-Seq analysis of −0.1 V versus standard hydrogen electrode (SHE) electrode-grown cells identified mutations in a subset of genes encoding cytochromes, processing systems for proline-rich proteins, sensory networks, extracellular structures, polysaccharides, and metabolic enzymes that caused at least a 50% reduction in apparent growth rate. Scarless deletion mutants of select genes identified via Tn-Seq revealed a new putative porin-cytochrome conduit complex (*extABCD*) crucial for growth with electrodes, which was not required for Fe(III) oxide reduction. In addition, four mutants lacking components of a putative methyl-accepting chemotaxis–cyclic dinucleotide sensing network (*esnABCD*) were defective in electrode colonization but grew normally with Fe(III) oxides. These results suggest that G. sulfurreducens possesses distinct mechanisms for recognition, colonization, and reduction of electrodes compared to Fe(III) oxides.

**IMPORTANCE** Since metal oxide electron acceptors are insoluble, one hypothesis is that cells sense and reduce metals using the same molecular mechanisms used to form biofilms on electrodes and produce electricity. However, by simultaneously comparing thousands of Geobacter sulfurreducens transposon mutants undergoing electrode-dependent respiration, we discovered new cytochromes and chemosensory proteins supporting growth with electrodes that are not required for metal respiration. This supports an emerging model where G. sulfurreducens recognizes surfaces and forms conductive biofilms using mechanisms distinct from those used for growth with metal oxides. These findings provide a possible explanation for studies that correlate electricity generation with syntrophic interspecies electron transfer by Geobacter and reveal many previously unrecognized targets for engineering this useful capability in other organisms.

## INTRODUCTION

While most electron acceptors are soluble and easily reduced by inner membrane respiratory proteins, some electron acceptors are insoluble and exist beyond the cell surface. Bacteria able to catalyze extracellular electron transfer or extracellular respiration contain redox proteins and attachment mechanisms able to create electrical connections between membranes and these external substrates. Multiple modes of extracellular respiration are known, including reduction of Fe(III) and Mn(IV) oxides (1, 2), electrical current production at electrode surfaces (3, 4), and formation of between-cell conductive networks enabling syntrophic growth with electron-consuming methanogens (5, 6).

The facultative anaerobe Shewanella oneidensis uses a single pathway composed of the CymA inner membrane cytochrome and the MtrCAB porin-multiheme *c*-type cytochrome complex to deliver electrons to all tested external metals and electrodes (7, 8). In contrast, there is evidence for multiple levels of complexity in the respiratory chain of the anaerobe Geobacter sulfurreducens. G. sulfurreducens requires two different inner membrane *c*-type cytochromes, ImcH and CbcL, depending on the reduction potential of the extracellular acceptor (9–11). Up to five homologs of the PpcA periplasmic triheme *c*-type cytochrome could then be involved in assisting electron transfer across the periplasm (12). To cross the outer membrane, one conductive porin-cytochrome “conduit” complex consisting of the OmaB multiheme *c*-type cytochrome, OmbB porin-like protein, and OmcB multiheme lipoprotein *c*-type cytochrome is linked to growth with some metals (13, 14), but the genome contains at least five other putative porin-cytochrome gene clusters with unknown functions. Beyond the outer surface, Geobacter must also build a conductive interface to reach metal particles, electrode surfaces, or partner bacteria. Extracellular polysaccharides and lipopolysaccharides influence surface adhesion and biofilm interactions (15), while a combination of conductive type IV pili and multiheme *c*-type cytochromes, such as OmcS, OmcE, OmcZ, and PgcA, are implicated in electron transfer beyond the outer membrane, depending on the growth conditions (13, 15–17).

Due to the ease of quickly screening mutants in liquid medium compared to growing mutants on electrodes in electrochemical reactors, components of the Geobacter electron transfer pathway have been identified using metals as proxies for extracellular acceptors (15). Transposon-based mutagenesis using metals revealed crucial cytochromes and attachment strategies, but high-throughput screens identify only the most severe defects, where mutant growth rates are near zero (18). Unfortunately, as multiple overlapping electron transfer pathways exist and many electron transfer proteins have functional homologs, single mutations typically decrease rather than eliminate the growth of G. sulfurreducens (19, 20). This respiratory complexity, combined with the bottleneck of studying electron transfer in electrode biofilms, has thwarted the use of unbiased mutagenesis-based discovery tools in the study of electricity production.

Transposon-insertion sequencing, also known as Tn-Seq, is able to quantify the abundance of every mutant containing a transposon insertion within a library, without growing individual mutants in isolation (21). Using Tn-Seq, it is possible to measure the effect every mutation has on the growth rate by comparing an insertion's abundance before and after a known number of generations. This method rapidly assessed gene essentiality under aerobic versus anaerobic growth in Shewanella oneidensis, revealed genes crucial to different respiration strategies in Rhodopseudomonas palustris, and uncovered the essential gene set of the archaeon Methanococcus maripaludis (22–24). For this report, we constructed the first G. sulfurreducens Tn-Seq libraries and discovered over 1,200 genes essential for growth in minimal medium with a soluble electron acceptor. By cultivating this same mutant library on electrode surfaces, a subset of mutations specifically involved in electrode colonization and respiration was revealed. Mutants reconstructed to test Tn-Seq data, such as those lacking new key cytochromes or chemosensory genes, were severely impaired in growth with the electrode. However, these electrode-deficient mutants remained fully able to respire extracellular metal acceptors, such as Fe(III) oxides, even though the redox potentials of electrodes and Fe(III) oxides used in these experiments were similar. These results confirm that electrode growth is a phenotype requiring previously unrecognized intracellular and extracellular components. In addition, these data show that while electrodes and environmental metals may be similar in terms of extracellular location and redox potential, separate biochemical mechanisms can be used to recognize and reduce these different acceptors.

## RESULTS AND DISCUSSION

### Tn-Seq in G. sulfurreducens.

G. sulfurreducens transposon libraries were generated using a mariner-based transposon with directional type IIS MmeI recognition sites engineered on both ends of the transposon insertion sequence (24, 25). As MmeI cleaves chromosomal DNA 20 bp from its recognition site, adapter ligation and amplification allow sequencing to identify insertions and quantify each mutant's abundance (26). After three separate transposon libraries were constructed using fumarate as the electron acceptor, approximately 50,000 individual colonies were recovered per library, and sequencing revealed between 30,000 and 33,000 independent insertions in each library with no obvious biases in coverage. The library representing the deepest coverage was used as the source for all experiments reported in this work.

**FIG 1:**
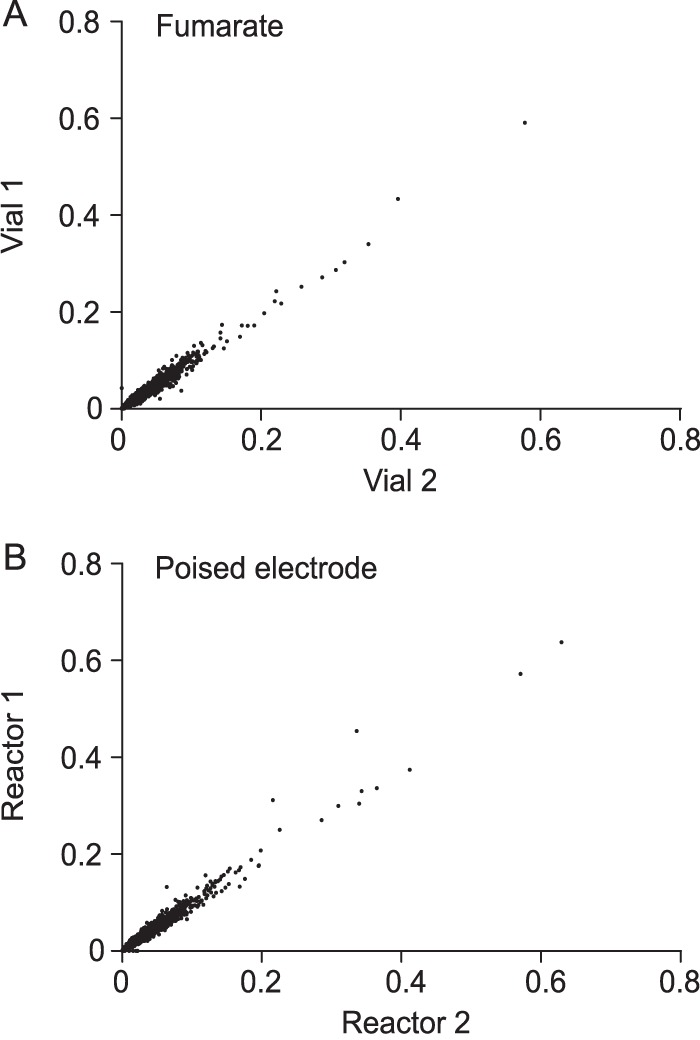
Tn-Seq is reproducible between library replicates and between experimental replicates in Geobacter sulfurreducens. (A) Comparison of two subsamples of the same library grown in two independent replicates with fumarate. The number of reads mapped to a gene (normalized for read depth) is plotted against reads mapped to the same gene for two experiments prepared and sequenced separately. (B) Comparison of two cultures inoculated separately in reactors a poised electrode as a terminal electron acceptor, recovered and sequenced separately. Both fumarate and poised electrode libraries have a Pearson correlation coefficient of 0.98.

When DNA from two separate cultures within the same library was extracted, digested with MmeI, ligated to Illumina adaptors, amplified, and sequenced, 33,257 and 33,343 unique insertions were identified in the two replicates. Over 97% of the insertions occurred within annotated features, at an average density of 9 unique insertions per kb. Despite opportunities for bottlenecking and bias during each growth, extraction, labeling, and sequencing step, the number of reads mapped per annotated feature was reproducible, with a Pearson's coefficient of 0.98 between the two fumarate culture replicates (Fig. 1A). When two independent cultures were further grown with poised electrodes as the electron acceptor and then separately recovered, extracted, and labeled, the number of reads mapped per gene in each independent growth experiment was similarly reproducible, with a Pearson's coefficient of 0.98 (Fig. 1B).

### Genes supporting growth using fumarate in G. sulfurreducens.

To predict genes important for growth with electrodes, genes required for growth in standard minimal medium with the electron acceptor fumarate were first determined so they could be excluded from further analyses. Tn-Seq libraries were grown in liquid culture for 6 generations and sequenced to a depth of >40 M reads per culture replicate. At this depth, an average of 1,200 reads are obtained per insertion, and based on an average feature size in G. sulfurreducens of 0.95 kb/feature, 10,200 reads are expected to map to each feature in the annotated genome.

**FIG 2:**
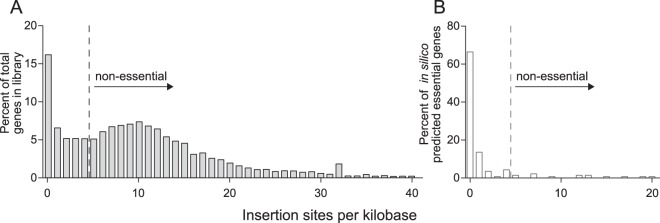
Estimation of essential genes based on low insertion densities and use of *in silico* model data to verify essentiality predictions. (A) The frequency distribution of insertions is centered around 10 insertions/kb, with enrichment below 4 insertions per kb (left of the dashed line). Genes with few insertions are predicted to be essential under these conditions. (B) Most genes labeled as essential in *in silico* modeling also contained fewer than 4 insertion sites per kb and had fewer than 300 mapped reads/gene (27).

Previous constraint-based modeling predicted 140 core metabolic and biosynthetic genes that should be important for growth using fumarate as the sole electron acceptor (27). The majority of these predicted core genes had fewer than 300 mapped reads in our Tn-Seq analysis, compared to an expected 10,200 reads/gene, and contained fewer than 4 insertions per kb (Fig. 2; also see Table S2 in the supplemental material). Genes predicted to be nonessential *in silico* averaged over 8,100 reads/feature and 10 insertions per kb. Based on these results and considering the depth of sequencing, annotated features with fewer than 300 reads mapped per feature or 4 insertion sites per kb were predicted to be important for laboratory growth. Using both criteria allowed for the correction for small genes containing only a few transposon insertion sites (TA dinucleotide), as well as larger genes which support insertions between domains (28). According to this cutoff, over 170 genes previously coded as nonessential in the *in silico* model were essential in our minimal medium, such as genes in biosynthetic pathways for biotin, riboflavin, and cobamide (as the test medium lacks vitamins). In addition, biosynthesis pathways for heme, fatty acids, and lipopolysaccharides, along with putative transporters for sulfate, acetate, copper, cobalt, and magnesium, were identified. Complete tables comparing *in silico* predictions with Tn-Seq results are in Tables S3 and S4.

Out of a total of 3,706 NCBI-annotated genome features, disruptions in 1,214 features had a major fitness defect during laboratory growth in liquid medium with acetate as the electron donor and fumarate as the sole electron acceptor. A key difficulty in the original G. sulfurreducens annotation and *in silico* metabolic model was that genes encoding homologs or redundant pathways were assumed to complement each other (18, 19). Tn-Seq analysis clarified roles for hundreds of such genes. For example, despite the presence of five annotated ferredoxins, transposon insertions in only one (GSU2708) caused a severe fitness defect, one of three 2-oxoglutarate dehydrogenase complexes was essential (GSU1467 to GSU1470), one of three aconitase enzymes was essential (GSU1660), one of two fructose bisphosphate aldolases was essential (GSU1193), and two of three phosphoglycerate mutase enzymes were essential (GSU1818 and GSU3207). In total, Tn-Seq resolved the essentiality of over 270 such examples where homologs were present but only specific genes were required under laboratory conditions (Tables S5 and S6).

Of special interest in Geobacter electron transfer is the mechanism of NADH oxidation and electron delivery to the quinone pool. This remains poorly understood, partially due to the fact that most Geobacteraceae encode two unrelated complex I-NADH dehydrogenases (29). The complex I gene cluster similar to that found in other proteobacteria, recently classified as type “E” (GSU3429 to GSU3445), was not required during fumarate growth. However, transposon insertions in a second less-well-characterized complex I caused major defects. This NADH dehydrogenase does not fall into a major defined clade and is most closely related to complex I in Chloroflexi (GSU0338 to GSU0351) (29). None of the genes in this NADH dehydrogenase operon had more than 300 mapped insertions or 4 insertion sites per kb. This large essential operon has only one known transcriptional start site, yet transposon insertions occurred throughout the intergenic regions, providing evidence that transcriptional readthrough from the transposon's kanamycin cassette, which lacks any transcriptional termination sequences, was sufficient to alleviate polar effects during the Tn-Seq analysis. (More examples showing the lack of polar effects in essential operons can viewed using the instructions in File S7.)

A second essential gene cluster revealed via Tn-Seq with significant bioenergetic implications was a 4-gene integral membrane *b*-type cytochrome-FeS cluster-electron transfer flavoprotein. These genes (GSU2795 to GSU2797) encode all signature residues of recently described confurcation-bifurcation complexes. This suggests that low-potential electron carriers, such as ferredoxin, could be used to help high-potential electron carriers, such as NADH, reduce the quinone pool via confurcation, or that bifurcation via ferredoxin oxidation linked to NAD^+^ reduction could be used to gain additional proton translocation during oxidation of acetate (30).

### Genes affecting electrode growth of G. sulfurreducens.

**FIG 3:**
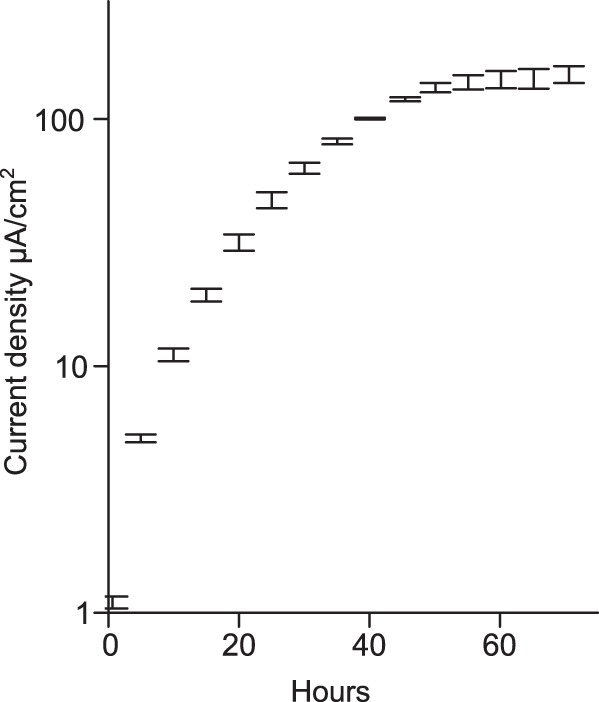
Replicate G. sulfurreducens Tn-Seq library biofilms grow at similar rates with similar current density. Replicate (*n* = 2) Tn-Seq libraries were inoculated independently into 3-electrode bioreactors, with anodes poised to mimic Fe(III) oxides (−0.1 V versus SHE). Biofilms were harvested after the equivalent of 6 doublings in biofilm growth mode. Error bars represent standard deviations.

Growth on an electrode surface in microbial fuel cells and electrochemical devices is hypothesized to be a phenotype that requires attachment, movement of electrons across membranes, and formation of between-cell conductivity to sustain respiration by cells not in contact with the surface (9, 10, 15, 31, 32). To test the entire process from attachment to biofilm growth, two separate Tn-Seq libraries were grown for the equivalent of 6 generations on electrodes poised at −0.1 V versus standard hydrogen electrode (SHE) (Fig. 3), and the density of reads mapped per feature was compared to parallel fumarate-grown cells. Because redox potential is known to affect the cytochromes needed for electron transfer, the electrode potential (−0.1 V versus SHE) was chosen to mimic the redox potential of environmental Fe(III) oxides (33). At this electrode potential, the replicate G. sulfurreducens Tn-Seq library biofilms had similar growth rates and final current densities, with current doubling at a maximum rate of every 10 h, similar to previous reports (9, 10).

Expressing Tn-Seq read density data as a log_2_ ratio allows a quick assessment of phenotypes. For example, a mutation preventing growth results in half as many reads mapping to that site after one generation, or a log_2_ ratio of −1 compared to the wild type. Using the exponential-growth equation, genes with a log_2_ score of −2 after 6 generations are equivalent to at least a 50% reduction in apparent doubling time during the experiment. Using this criterion, at least 39 genes encoding cytochromes, protein processing systems, sensing proteins, extracellular structures, polysaccharides, metabolic enzymes, and hypothetical proteins were involved in electrode growth. For future use by the community, the full data set showing all read mapping densities and log_2_ ratios for all features is in Table S2, with indications if genes were previously judged as nonessential under fumarate-grown conditions. Separate tables (Tables S3 to S6) are provided that process subsets of data, such as comparisons to previously predicted essential genes in the *in silico* model. Direct links for downloading our read mapping files and the annotated genome needed for viewing in software such as IGV are in File S7, along with instructions for viewing the results.

Tn-Seq analysis confirmed previous findings that CbcL, an inner membrane putative quinone oxidoreductase containing both *b*- and *c*-type cytochromes (GSU0274), is crucial when electrodes are poised at −0.1 V versus SHE (9, 10). Insertions in *cbcL* resulted in a log_2_ ratio corresponding to doubling time of 17.4 h, while a 20-h doubling time was previously observed for a pure-culture Δ*cbcL* scarless deletion mutant (9). Also in agreement with previous work was the severe negative impact of insertions in genes involved in assembly and expression of the conductive type IV pili. Insertions in the *pilQPOM* operon (GSU2028 to GSU2032), *pilC* gene (GSU1493), and *pilS* sensor kinase (GSU1494) produced similar defects, while insertions in other type IV pilus genes, such as *pilA* (GSU1496), predicted weaker phenotypes (below the threshold in Table 1), but still suggesting at least a 25% decrease in apparent growth rate. Insertions in some pilus genes, such as the *pilT4* gene (GSU1492), led to increased abundance, consistent with recent reports that *pilT4* mutants more rapidly colonize electrodes (34).

**TABLE 1:** Tn-Seq mutations which after growth on an electrode showed a decrease in reads mapped by at least a log_2_ ratio of −2, equivalent to a predicted 50% reduction in growth rate

Locus	Gene symbol and/or product description	Log_2_ ratio
Cytochrome		
GSU0274	*cbcL*, *c*- and *b*-type cytochrome	−3.3
GSU2643	*extC*, lipoprotein cytochrome *c*	−2.0
GSU2645	*extA*, cytochrome *c*	−2.5
Metabolism and protein processing		
GSU0140	Phosphoribosylaminoimidazole carboxylase-like protein	−2.2
GSU0503	*crcB* integral membrane protein, putative transporter	−2.2
GSU0536	Adenosine nucleotide alpha-hydrolase superfamily protein	−2.6
GSU0994	*fumB*, fumarate hydratase	−8.0
GSU1105	Prolidase family protein	−2.0
GSU1279	*nikMN*, nickel ABC transporter membrane protein NikMN	−2.0
GSU1752	*efp-2*, elongation factor P	−2.5
GSU1753	*genX*, translation elongation factor P-lysine lysyltransferase	−2.9
GSU1754	*yjeK*, translation elongation factor P-lysyl-lysine 2,3-aminomutase	−2.8
GSU3278	Outer membrane TPR-containing protein^*a*^	−2.0
Signaling and regulation		
GSU0013	MarR family winged helix-turn-helix transcriptional regulator	−2.1
GSU1704	*esnA*, GAF sensor methyl-accepting chemotaxis sensory transducer, class 40H	−2.4
GSU2220	*esnB*, scaffold protein CheW associated with MCPs of class 40H	−2.4
GSU2221	ATPase	−2.1
GSU2222	*esnC*, sensor histidine kinase CheA associated with MCPs of class 40H	−2.8
Extracellular structures		
GSU1114	Lipoprotein	−2.1
GSU1493	*pilC*, type IV pilus inner membrane protein PilC	−2.2
GSU1494	*pilS*, sensor histidine kinase PilS, PAS domain containing	−2.2
GSU1501	*xapD*, ABC transporter ATP-binding protein	−2.0
GSU1816	*ugd*, UDP-glucose 6-dehydrogenase	−2.0
GSU1889	*lptA*, lipopolysaccharide ABC transporter periplasmic protein LptA	−6.5
GSU1976	YqgM-like family glycosyltransferase	−4.9
GSU2028	*pilQ*, type IV pilus secretin lipoprotein PilQ	−2.5
GSU2029	*pilP*, type IV pilus assembly lipoprotein PilP	−3.3
GSU2030	*pilO*, type IV pilus biogenesis protein PilO	−2.5
GSU2032	*pilM*, type IV pilus biogenesis ATPase PilM	−2.4
GSU2085	*hldE*, d-glycero-d-mannoheptose-7-phosphate kinase and d-glycero-d-mannoheptose-1-phosphate adenylyltransferase	−3.1
GSU2086	Hypothetical cytoplasmic protein in sugar biosynthesis operon	−3.2
GSU2087	*gmhA*, phosphoheptose isomerase	−3.7
GSU2257	Hypothetical cytoplasmic protein in LPS biosynthesis operon	−2.4
GSU2973	Lipoprotein	−2.7
GSU3321	Phosphoglucomutase/phosphomannomutase family protein	−2.2
Hypothetical		
GSU0141	Hypothetical cytoplasmic protein	−2.3
GSU0959	Hypothetical protein	−2.3
GSU2048	Hypothetical protein	−2.1
GSU2713	Hypothetical protein (chaperone-like protein)	−2.9

a TPR, tetratricopeptide repeat.

With the exception of CbcL, no other cytochromes previously reported to play a role in electron transfer to metals appeared to be required for growth with an electrode poised at −0.1 V versus SHE (Table 1). Instead, insertions in two uncharacterized multiheme *c*-type cytochromes (GSU2643 and GSU2645) significantly affected electrode growth. These cytochromes are part of a larger cluster containing two multiheme *c*-type cytochromes, a multiheme lipoprotein cytochrome, and a predicted outer membrane porin-like protein. As a similar arrangement of multiheme cytochromes, lipoprotein cytochromes, and putative β-barrel proteins encodes what has recently been termed a “conduit” for electron transfer across the outer membrane in both Geobacter (the OmcB-based conduit) and Shewanella (the MtrCAB conduit), this region was targeted for further study, described in the next section.

The G. sulfurreducens genome encodes over 100 histidine kinases, response regulators, and chemotaxis-like proteins, yet little is known about their role in extracellular respiration (35). Tn-Seq identified one DNA-binding protein (GSU0013) and four chemotaxis proteins (GSU1704 and GSU2220 to GSU2222) as significant. Only one diguanylate cyclase response regulator was suggested to participate in electrode-dependent growth (GSU3376) with at least a predicted 25% decrease in growth rate. In model systems, methyl-accepting chemotaxis proteins, such as GSU1704, undergo a conformational change to interact with CheW (GSU2220) and CheA (GSU2222) family proteins, triggering phosphorylation of GSU3376-like response regulators (36). Based on the hypothesis that GSU1704, GSU2220, GSU2222, and GSU3376 represented portions of the MCP-CheA-CheW (MCP, methyl-accepting chemotaxis-like protein) response regulator systems involving the biofilm regulator cyclic di-GMP (c-di-GMP), these were also targeted for further study, as described in the next section.

Some of the strongest phenotypes in apparent electrode growth were due to insertions in genes encoding sugar and polysaccharide synthesis. Five different sugar dehydrogenases, sugar transferases, and isomerases were identified as being required for electrode growth via Tn-Seq, and insertions within three different gene clusters involved in capsule, lipopolysaccharide, and extracellular sugar synthesis produced significant growth defects. Also crucial were genes putatively involved in translocating sugars to the outer surface, such as the gene for a sugar ABC transporter previously shown to be essential for electrode growth (GSU1501, *xapD*), and the gene for a lipopolysaccharide (LPS) ABC transporter protein (GSU1889, *lptA*). The defects created by these insertions further suggest a role for a properly charged or modified outer surface during Geobacter biofilm formation on electrodes (15, 17).

Finally, proteins involved in translation and processing of proline-rich proteins became important during electrode growth. For example, elongation factor P (EF-P) is important for overcoming ribosome stalling during translation of proline-repeat sequences and contributes to outer membrane integrity in Escherichia coli (37, 38). EF-P is essential in many bacteria and is required for swarming and surface motility in Bacillus subtilis and pathogenesis in Salmonella enterica, Agrobacterium tumefaciens, and Pseudomonas aeruginosa (39–43, 73). Tn-Seq analysis predicted that one of two EF-P homologs in G. sulfurreducens (GSU1752, *efp-2*), along with the EF-P-modifying EF-P lysine-lysyltransferase (GSU1753) and EF-P lysyl-lysine 2,3-aminomutase (GSU1754), became important during growth with electrodes. Insertions in a prolidase involved in cleaving peptides at proline residues (GSU1105) and peptidylprolyl *cis-trans* isomerase (GSU2074) showed strong growth defects, further suggesting an unrecognized importance in the folding and processing of proline-rich proteins under electrode-respiring conditions.

### Possible genes missed by the community aspect of Tn-Seq analysis.

Deletion of the outer surface cytochrome gene *omcZ* (GSU2076) can significantly decrease electrode growth (44). While Tn-Seq insertions in most genes within the *omcZ* operon had a negative impact (such as the peptidylprolyl *cis-trans* isomerase, GSU2074), insertions in *omcZ per se* were not identified as causing a defect in library experiments (44, 45). Similarly, the pilus-associated cytochrome OmcS is involved in electron transfer to electrodes, yet it was not identified as above the threshold in our analysis. Both of these cytochromes are verified to exist beyond the cell membrane in the conductive matrix between cells (45, 46).

Under Tn-Seq conditions, extracellular proteins and soluble metabolites can be shared. A clear example of population-based complementation is evident in Tn-Seq data for fumarate hydratase (GSU0944), encoded by a tricarboxylic acid (TCA) cycle gene predicted to be absolutely essential (Table S3). As wild-type G. sulfurreducens secretes malate when grown with fumarate (47), extracellular malate produced by wild-type cells can rescue a gap in the TCA cycle of rare fumarate hydratase mutants. Consistent with this hypothesis, during growth with an electrode in the absence of fumarate, the fumarate hydratase gene emerged as one of the most important genes in Tn-Seq analysis.

These data suggest that extracellular cytochromes, such as OmcZ, secreted by wild-type cells may similarly aid the occasional *omcZ::*mini*Himar* mutant in a biofilm population. This phenomenon would be similar to how outer membrane proteins shared between Myxococcus strains rescue motility mutants (48, 49), and siderophores act as “public goods” for rare nonproducing strains (50). In contrast to evidence for OmcS and OmcZ sharing between cells, cellular machinery, such as the type IV pilus apparatus, inner membrane cytochromes, and outer membrane cytochromes remained crucial even under biofilm conditions. Future experiments combining low levels of mutants lacking extracellular proteins with the wild type could show which Geobacter proteins can be shared for communal conductivity and exploited by “cheaters” embedded in a conductive matrix, compared to which proteins catalyze key attachment or electron transfer reactions so essential that they cannot be borrowed from neighbors.

### Cytochrome conduit deletion mutants affect electrode reduction but not Fe(III) reduction.

While Tn-Seq agreed with the importance of previously reported mechanisms, such as pili, inner membrane cytochromes, and extracellular sugars, it identified many processes never highlighted in any mutant, proteomic, or transcriptional study. Based on their strong phenotypes predicted by Tn-Seq, we further investigated two classes of mutants, involving new outer membrane cytochromes and signaling proteins by constructing scarless deletions of these key genes and gene clusters.

In the Tn-Seq data, little effect was observed from mutations in the highly abundant, well-studied outer membrane *c*-type cytochrome OmcB (51). OmcB is encoded in part of a three-gene cluster (*ombB-omaB-omcB*, GSU2737 to GSU2739) next to a nearly identical tandem duplication containing another cytochrome–porin-like protein–lipoprotein cytochrome conduit (*ombC-omaC-omcC*, GSU2731 to GSU2733). This duplication could have limited the impact of single mutations during Tn-Seq. To more definitively test if OmcB family conduits were involved in electrode respiration, a scarless deletion of this entire genomic region lacking all genes in the *omcB*- and *omcC*-encoded conduits was constructed, and it is referred to here as Δ*omcBC* (ΔGSU2739 to GSU2731).

Genes for a different putative porin-cytochrome conduit not homologous to the OmcB or OmcC conduit showed a strong phenotype in the Tn-Seq data (Table 1). We referred to genes in new clusters containing genes for multiple cytochromes, beta-barrels, iron-sulfur proteins, and lipoproteins as *ext* regions (extracellular electron transfer), starting with the region linked to the strongest phenotype as *extABCD*. All four genes were removed to generate the Δ*extABCD* (ΔGSU2645 to GSU2642) mutant strain. In addition, two other putative outer membrane conduit clusters, the Δ*extEFG* (ΔGSU2726 to GSU2724) and Δ*extHIJKL* (ΔGSU2940 to GSU2936) mutants, were constructed for comparison with Tn-Seq results.

**FIG 4:**
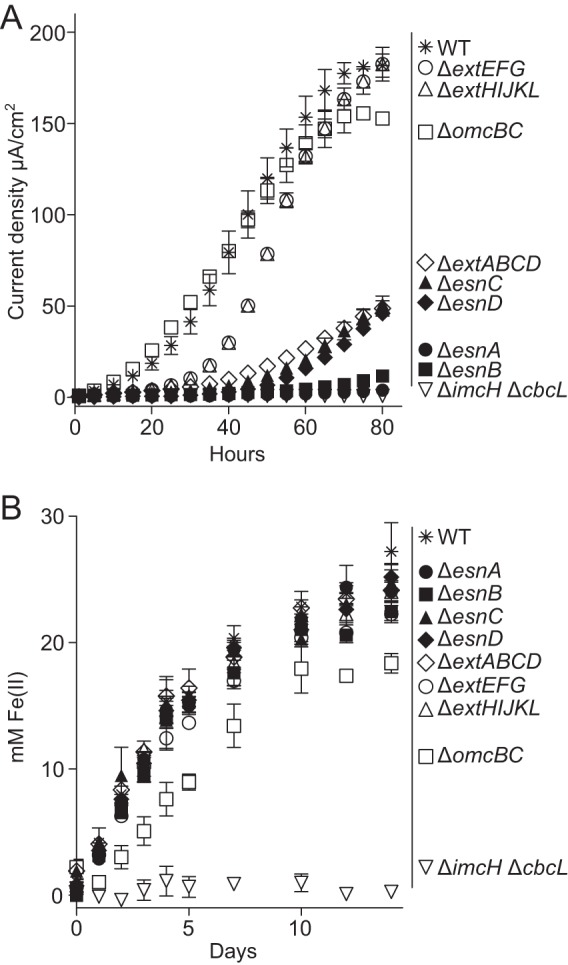
Genes essential for growth with electrodes are not required for Fe(III) reduction. (A and B) Growth of scarless deletion mutants of chemosensory genes *esnA*, *esnB*, *esnC*, and *esnD*, and extracellular conduit clusters *extABCD*, *omcBC*, *extEFG*, and *extHIJKL* with poised electrodes (A) or insoluble Fe(III) oxides (B). All incubations or reactor experiments were performed for each strain in triplicate. A mutant lacking both inner membrane cytochromes important for using extracellular acceptors (Δ*cbcL* Δ*imcH*) was used as a negative control. Representative curves are from replicates in all growth experiments. Error bars represent standard deviations.

The Δ*extABCD* mutant strain grew poorly when the electrode was the electron acceptor, never producing more than 50 μA/cm^2^ when grown under the same conditions used for Tn-Seq (electrodes poised at −0.1 V versus SHE). In contrast, the Δ*omcBC* mutant strain produced 80% of the wild-type (WT) current densities at 80 h (Fig. 4A). The other mutants lacking outer membrane conduits, the Δ*extEFG* and Δ*extHIJKL* mutants, also showed minimal defects on the electrode. All conduit mutants grew at wild-type growth rates using fumarate as the electron acceptor, as predicted by the Tn-Seq data. While single-gene replacement mutants lacking the *omcB* gene are known to grow in microbial fuel cells (52), these results indicate that no part of the *ombB-omaB-omcB* or *ombC-omaC-omcC* conduit genes is required for reduction of electrodes, and two other putative trans-outer membrane conduit gene clusters (*extEFG* and *extHIJKL*) are also not required for electron transfer to low-potential electrodes. In both Tn-Seq and pure-culture studies with reconstructed mutants, the deletion of *extABCD* had a significant effect only when electrodes were the electron acceptor. Further phenotypic analysis with electrodes poised at higher reduction potentials is needed to test the specificity of the ExtABCD conduit under all electrode growth conditions (9–11).

Even though the metal oxide particles were surfaces with redox potentials similar to those of the electrodes used in this Tn-Seq analysis, the *extABCD* gene cluster was not required for the reduction of Fe(III) oxides (Fig. 4B). This was in contrast to the strong electrode phenotype observed for the mutant lacking *extABCD*. The markerless Δ*omcBC* mutant strain exhibited a Fe(III) oxide reduction defect, while the Δ*extEFG* and Δ*extHIJKL* conduit mutant strains had Fe(III) oxide reduction rates similar to the wild type. A Δ*imcH* Δ*cbcL* mutant lacking both inner membrane cytochromes was unable to reduce Fe(III) oxide (Fig. 4B). The phenotype of these mutants suggests that the dominant expressed pathway used for electron transfer across the outer membrane can vary, based on what extracellular electron acceptor is available.

### Chemosensory system deletion mutants also affect electrode reduction but not Fe(III) reduction.

A second class of genes revealed by Tn-Seq analysis were related to intracellular sensing and signaling systems. Based on their phenotypes during the electrode-based screen, these proteins were tentatively named part of an electrode sensing network (Esn) and included EsnA, an MCP (GSU1704); EnsB, a CheW-like putative scaffolding protein (GSU2220); EsnC, a CheA-like histidine kinase (GSU2222); and EsnD, a diguanylate cyclase (GSU3376). Scarless deletion mutants of each *esn* gene were constructed and tested for growth using an electrode as the electron acceptor.

All four *esn* deletion mutants displayed severe defects in growth with an electrode, as predicted by Tn-Seq data (Fig. 4A and Table 1). However, when these same four *esn* mutants were grown with Fe(III) oxide as the electron acceptor, Fe(III) reduction was similar to that of the wild type (Fig. 4B). All mutants also grew at wild-type growth rates with fumarate as the electron acceptor. This phenotype, where a mutant performed poorly with an electrode surface but still reduced metal oxide particles, agreed with the hypothesis that cells interact with these two solid-phase electron acceptors via different molecular mechanisms.

In a chemosensory complex, a methyl-accepting chemotaxis protein dimerizes and associates with both scaffolding and histidine kinase proteins for signal transduction to a downstream response regulator. While the Δ*esnA*, Δ*esnB*, and Δ*esnC* mutant strains shared similar phenotypes, *esnA* was not in the same gene cluster as *esnBC*. To test the hypothesis that EsnABC forms a complex, a bacterial two-hybrid assay was conducted using EsnA as bait and either the EsnA, EsnB, or EsnC protein as prey. As shown in Table 2, all three of these proteins showed positive results for EsnA association, consistent with a possible EsnABC complex (Table 2). Further *in vitro* protein-protein interaction experiments will be needed to confirm the specificity of this complex.

**TABLE 2:** Evidence for EsnABC association via two-hybrid analysis^*a*^

Coexpression with pEsnA bait	LacZ activity (ΔOD_420_ min^−1^ · OD_600_^−1^)
pSR658 (negative control)	1,378.5 ± 131.8
pEsnA prey (EsnA-EsnA)	287.9 ± 15.6
pEsnB prey (EsnA-EsnB)	473.6 ± 44.2
pEsnC prey (EsnA-EsnC)	448.3 ± 46.5

a LacZ activity is calculated based on the results from biological triplicates ± standard deviations. Lower LacZ activity indicates stronger protein-protein associations, causing stronger repression.

The EsnA methyl-accepting domain-containing protein has no putative periplasmic domain to suggest an extracellular input, but EsnA does contain a cytoplasmic GAF domain typically involved in sensing small molecules, such as cyclic nucleotides. The response regulator important for electrode growth, EsnD, recently was shown to act as a cyclic di-GMP synthase (53). One hypothesis is that EsnABCD regulate and reinforce the accumulation of cyclic di-GMP, driving the switch to a committed conductive biofilm mode of growth (54). More work is required to confirm the specificity of EsnABCD for an electrode versus general biofilm mode of growth, but a previous transposon study did not identify disruptions to the genes encoding these proteins as causing an attachment defect to 96-well plates (18). Regardless of the ultimate signal for electrode colonization, EsnABCD are not essential sensory proteins for growth with Fe(III) oxides.

### Conclusions and outlook.

**FIG 5:**
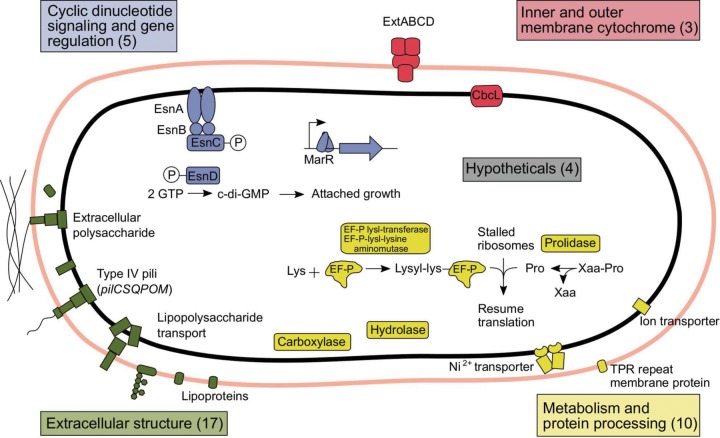
Genes involved in electrode reduction fall into four main categories. Of all genes identified under Tn-Seq conditions as causing a >50% reduction in growth rate on electrodes, most appeared to be involved in modifying sugars on the external surface of the cell, producing the pilus secretion apparatus, or modifying protein translation inside the cell. At −0.1 V versus SHE, only the CbcL inner membrane cytochrome and the ExtABCD porin-cytochrome conduit were essential. The only two regulatory systems identified involved cyclic di-GMP signaling and a previously unstudied MarR family DNA-binding protein.

The ability of Tn-Seq to survey mutants across the entire genome in a single experiment is especially useful when no high-throughput assay is available for the phenotype under study. In the case of growth on electrodes, given the growth rate of G. sulfurreducens, testing 33,000 individual mutants in replicate electrode reactors would require our laboratory to dedicate its bank of 45 electrochemical cells to the testing of mutants nonstop for over 28 years. In a fraction of that time, saturation mutagenesis predicted over 1,200 genes essential for growth under laboratory conditions, resolved hundreds of annotation issues, and brought into focus a subset of genes vital under electrode growth conditions at reduction potentials that are closer to those of environmentally relevant Fe(III) oxides (Fig. 5).

By asking what genes are required for growth, Tn-Seq generates data different from expression-based approaches that operate under the hypothesis that important genes will be strongly regulated in response to an electrode. In general, we found little overlap between genes predicted to be important to growth on electrodes via Tn-Seq and those up- or downregulated in expression or proteomic studies on electrodes (32, 44, 51). One possibility is that crucial regulatory or biosynthetic elements have low basal expression levels. Abundance-based studies could also have reduced sensitivity if biofilms are heterogeneous or if expression varies with distance from the electrode (55). In the case of cytochromes, the *imcH*- and *cbcL*-encoded cytochromes involved in electron transport are now known to be expressed even when cells are grown with fumarate, preventing their discovery via differential expression (9, 10). It also remains possible that electrodes, being relatively unnatural substrates, trigger transcriptional changes or derepress genes that have little to do with electrode respiration. Tn-Seq analyses with different extracellular acceptors, such as Fe(III) oxides, Mn(IV) oxides, and electrodes poised at other redox potentials likely uncover further specific gene sets crucial for their respiration.

Tn-Seq data provided genome scale information that agreed with prior *in silico* predictions and knockouts during fumarate reduction (27, 56). However, much less data are available for electrodes in terms of what genes are important, and as with any large-scale survey, reconstruction of mutants was needed to verify findings. Using scarless mutants lacking single genes and whole-gene clusters, we were able to confirm that individual chemosensory components of *esnABCD* and the entire cytochrome conduit *extABCD* identified in Tn-Seq were important when the electrode acted as the electron acceptor. As antibiotic insertion mutants in genes for outer membrane conduits based on OmcB and OmcC have defects in electron transfer to metals, we also constructed new markerless mutants lacking entire cytochrome clusters, along with mutants lacking two other unstudied putative outer membrane conduits. Surprisingly, only removal of the *extABCD* conduit gene cluster caused significant growth defects on electrodes, as predicted by initial Tn-Seq data. The reconstructed mutants act as verification of Tn-Seq results, and further genetic analysis of these clusters, such as knockouts of individual *ext* genes and complementation studies, will help elucidate the role of these genes in electron transfer.

When Fe(III) was the electron acceptor, none of the cytochrome conduit mutants with electrode phenotypes and none of the mutants lacking methyl-accepting chemotaxis proteins or GGDEF domain response regulators had significant defects (Fig. 4B). These data support a model that G. sulfurreducens possesses separate mechanisms for reduction of electrodes compared to the reduction of metals that involves a distinct set of cytochromes crossing the outer membrane. In addition, separate cyclic dinucleotide-dependent regulatory systems may be used for recognition and biofilm-dependent respiration of electrodes compared to interactions with metals.

A recent comparison of seven Geobacter species noted a poor correlation between electricity production and Fe(III) reduction rates (57). In contrast, strains capable of high rates of electricity production were also capable of interspecies electron transfer to methanogens. Along with the observation that anaerobic digesters rather than Fe(III)-rich environments remain the most common source of high-current-producing Geobacter enrichments (57–59), we propose that the conductive network used for laboratory electrodes may have evolved to support electron transfer between Geobacter and organisms, such as methanogens. A syntrophic partner may be much like an electrode: it can accept electrons indefinitely but requires long-term commitment to a biofilm lifestyle and the expense of secreting proteins to build a conductive extracellular space (6, 60). In contrast, small metal oxide particles represent a temporary and more variable electron acceptor, where cells cannot attach permanently to grow into thick biofilms that entrap and reuse expensive extracellular proteins (61). According to this view, metal oxides should not trigger a communal biofilm mode but rather a more independent temporary attachment lifestyle.

Naturally occurring Geobacter strains produce >1 mA/cm^2^ of electrical current, a rate that already rivals some of the best artificial enzyme-functionalized electrodes (62), but microbial electrochemistry as an energy source or biocatalyst is estimated to require at least a 10-fold increase in current density to be profitable (63–65). Achieving such gains and engineering new abilities into industrial bacteria first require identification of the core components essential to electricity production. The discovery of a distinct set of proteins required for electrode growth raises new questions regarding how organisms discriminate between extracellular Fe(III) oxides and electrodes, but it also greatly simplifies future work aimed at enhancing performance under specific conditions.

## MATERIALS AND METHODS

### Growth and medium conditions.

All strains and plasmids used in this study are listed in Table 3. G. sulfurreducens strains and mutants were grown from single-colony picks streaked from frozen dimethyl sulfoxide (DMSO) stocks in anoxic basal medium, as described previously (66). For routine growth, basal medium with acetate (20 mM) as the electron donor and fumarate (40 mM) as the electron acceptor were used. Agar (1.5%) was added to the acetate-fumarate medium to culture for clonal isolates on semisolid surface in a H_2_-CO_2_-N_2_ (5:20:75) atmosphere in a vinyl anaerobic chamber (Coy) or an anaerobic workstation 500 (Don Whitley). All growth analyses were initiated with a single colony on acetate-fumarate agar from freshly streaked frozen culture stocks. When electrodes were used as the electron acceptor, fumarate was replaced with 50 mM NaCl to maintain a similar ionic strength.

**TABLE 3:** Strains and plasmids used in this work

Strain or plasmid	Description or relevant genotype	Source or reference
Strains		
G. sulfurreducens		
DB790	Δ*cbcL* Δ*imcH* (ΔGSU0274 ΔGSU3259)	This study
DB823	Δ*esnA* (ΔGSU1704)	This study
DB836	Δ*esnB* (ΔGSU2220)	This study
DB824	Δ*esnC* (ΔGSU2222)	This study
DB1130	Δ*esnD* (ΔGSU3376)	This study
DB1280	Δ*extABCD* (ΔGSU2645 to GSU2642)	This study
DB1282	Δ*extEFG* (ΔGSU2726 to GSU2724)	This study
DB1279	Δ*omcBC* cluster (ΔGSU2739 to GSU2731)	This study
DB1281	Δ*extHIJKL* (ΔGSU2940 to GSU2936)	This study
E. coli		
S17-1	*recA pro hsdR RP4-2-Tc*::Mu-Km::Tn*7*	72
SU202	*lexA71*::Tn*5*(Def)*sulA211* Δ(*laclPOZYA*) 169/F′ *lacl*^q^ *lacZ*ΔM15::Tn*9*	71
BW29427 (WM3064)	*thrB1004 pro thi rpsL hsdS lacZ*ΔM15 *RP4-1360* Δ*(araBAD)567* Δ*dapA1341*::[*erm pir*]	K. Datsenko and B. L. Wanner
Plasmids		
pEB001	Plasmid carrying mini-*Himar* RB1 transposon with engineered MmeI restriction sites	24
pK18mobsacB	SacB-encoding scarless deletion vector	72
pDGSU0274	Flanking regions of GSU0274 in pK18mobsacB	9
pDGSU3259	Flanking regions of GSU3259 in pK18mobsacB	66
pDGSU1704	Flanking regions of GSU1704 in pK18mobsacB	This study
pDGSU2220	Flanking regions of GSU2220 in pK18mobsacB	This study
pDGSU2222	Flanking regions of GSU2222 in pK18mobsacB	This study
pDGSU3376	Flanking regions of GSU3376 in pK18mobsacB	This study
pDGSU2645-2642	Flanking regions of GSU2645 to GSU2642 in pK18mobsacB	This study
pDGSU2726-2724	Flanking regions of GSU2726 to GSU2724 in pK18mobsacB	This study
pDGSU2739-2731	Flanking regions of GSU2739 to GSU2731 in pK18mobsacB	This study
pDGSU2940-2936	Flanking regions of GSU2940 to GSU2936 in pK18mobsacB	This study
pSR658	Bacterial two-hybrid vector, WT LexA DNA-binding domain	71
pSR659	Bacterial two-hybrid vector, variant LexA DNA-binding domain	71
pEsnA-4 (bait)	pSR659 expressing the soluble domain of EsnA fused to variant LexA DNA-binding domain	This study
pEsnA-6 (prey)	pSR658 expressing the soluble domain of EsnA fused to WT LexA DNA-binding domain	This study
pEsnB-2 (prey)	pSR658 expressing EsnB fused to WT LexA DNA-binding domain	This study
pEsnC-2 (prey)	pSR658 expressing EsnC fused to WT LexA DNA-binding domain	This study

When Fe(III) oxide was used as the electron acceptor, a nonchelated mineral mixture was used (in which all components of the chelated mixture were dissolved in a small volume of 1 N HCl without nitrilotriacetic acid [NTA] [66]), as well as the following: 1.66 g/liter Na-acetate, 0.38 g/liter KCl, 0.2 g/liter NH_4_Cl, 0.6 g/liter NaH_2_PO_4_·H_2_O, 0.04 g/liter CaCl_2_·2H_2_O, and 0.2 g/liter MgSO_4_·7H_2_O. Medium with freshly precipitated β-FeO(OH) [here referred to as Fe(III) oxide] as the electron acceptor was prepared by precipitating Fe(III) from a 0.4 M FeCl_3_ solution by titrating with 25% NaOH at a rate of 1 ml per minute until the pH reached 7. This Fe(III) oxide was washed 3 times with deionized H_2_O before resuspending into half the original volume with deionized H_2_O. Fe(III) oxide growth medium was prepared by diluting the freshly precipitated Fe(III) oxide preparation 1:10 with the salt and mineral solution described above. The concentration of Fe(III) is roughly 55 mM in this medium, and recent work has shown the redox potential of this mineral preparation to be approximately −0.1 V versus SHE following autoclaving (11). In all cases, the pH of the medium was adjusted to 6.8, buffered with 2 g/liter NaHCO_3,_ and purged with N_2_:CO_2_ gas (80:20) passed over a heated copper column to remove trace oxygen.

Three-electrode bioreactors were assembled as previously described (67). Briefly, graphite electrodes were polished using 1,500-grit wet/dry sandpaper and attached to platinum wire to serve as the working electrode. A bare platinum wire was the counterelectrode. The potential of the working electrode was maintained at −0.10 V versus standard hydrogen electrode (SHE) using a saturated calomel reference electrode and a VMP3 multichannel potentiostat (Biologic). Reactor headspace was degassed using 80:20 N_2_:CO_2_ prior to inoculation. The current was measured as an average over 2 min. For all pure-culture mutant experiments, a single colony was inoculated into acetate-fumarate medium until the culture reached late exponential phase (optical density at 600 nm [OD_600_], 0.50), and then a 25% inoculum was used as the cultures reached acceptor limitation. The total volume of each reactor was 15 ml, and the working electrode surface area was 3 cm^2^.

To assay Fe(III) oxide reduction in G. sulfurreducens, a single colony was inoculated into acetate-fumarate liquid medium and grown to late exponential phase (OD_600_, 0.5). Minimal medium containing 20 mM acetate as the electron donor and 55 mM Fe(III) oxide as the sole electron acceptor was inoculated 1:100 from the acetate-fumarate-grown colony pick culture. A small sample (0.1 ml) of the medium was removed at regular intervals and dissolved in 0.5 N HCl for at least 24 h in the dark. The acid-extractable Fe(II) was measured using a modified FerroZine assay (66).

Escherichia coli was cultivated in lysogeny broth supplemented with 0.3 M 2,3-diaminopimelic acid and 50 μg/ml kanamycin when needed.

### Tn-Seq library construction.

Five milliliters of mid-log (OD_600_, ∼0.35) G. sulfurreducens culture grown from a single colony on acetate-fumarate was combined with 5 ml of an overnight culture of E. coli conjugative donor strain BW29427 (WM3064) carrying the transposon plasmid pEB001 was applied to a nitrocellulose filter (0.4-μm pore size) using a vacuum. Plasmid pEB001 contains the mariner derivative *Himar1* transposon with MmeI recognition sites on both sides of the inverted repeats that flank a kanamycin resistance cassette. A G. sulfurreducens recipient and E. coli donor mixture was washed on the filter with 3 volumes of basal medium before transferring the filter with the cell mixture to an agar plate containing acetate-fumarate and incubating in a Coy chamber at 30°C for 4 h. The cell mixture was washed off the filter disc with 1 ml of basal medium supplemented with 200 μg/ml kanamycin. To select for G. sulfurreducens transposon mutants, dilutions were plated on large 22- by 22-cm square agar plates with acetate-fumarate medium supplemented with kanamycin and incubated at 30°C in the anoxic chamber until visible colonies formed (in 6 days). Approximately 50,000 colonies were pooled in 10 ml of acetate-fumarate medium and grown for 4 h before freezing 1-ml aliquots in 10% DMSO.

### Tn-Seq experiment.

For each experiment, a frozen library aliquot was used to inoculate 100 ml of acetate-fumarate medium. When cultures reached exponential-growth phase (OD_600_, ∼0.35), 5 ml of this culture was inoculated into fresh 100 ml of acetate-fumarate medium. This parent culture was grown to early stationary phase (OD_600_, ∼0.5) and used to initiate both the electrode and fumarate control experiments. Five milliliters of the parent culture was inoculated into 100 ml of acetate-fumarate medium and allowed to grow for 6 generations before harvesting. At the same time, 7.5 ml of the parent culture was inoculated into the 3-electrode bioreactor containing 7.5 ml of medium, where two 3-cm^2^ working electrodes were present to support biofilm growth. G. sulfurreducens electrode biofilms were harvested by moving reactors to an anaerobic chamber after 6 generations of growth using electrodes poised at −0.1 V SHE, with the number of generations estimated based on the doubling time (10 h) at this redox potential. The entire electrode plus attached biofilm was placed in 100 ml of acetate-fumarate minimal medium, vortexed to liberate cells, and incubated at 30°C for 6 generations before harvesting. This outgrowth was necessary to produce adequate free biomass for DNA extraction and sequencing and is why cells grown with fumarate for an additional 6 generations were used as a parallel control. Despite this additional outgrowth step, the read densities and phenotypes between replicate experiments were highly repeatable (Fig. 1B). In replicate 50,000 clone libraries, any off-site mutation that can result in a growth benefit or defect is unlikely to mislead downstream results, due to saturation causing each gene to multiple independent mutants in the library, and the small number of doublings allowed.

Genomic DNA from 40 ml of cells was isolated using the Wizard genomic DNA purification kit (Promega). The protocol for preparing the DNA library for Illumina sequencing was outlined previously, with modifications described below (68). Six micrograms of genomic DNA (gDNA) was digested with MmeI (New England BioLabs) for 2 h at 37°C. To this reaction, Antarctic phosphatase (New England BioLabs) was added and incubated at 37°C for an additional hour. Enzymes were inactivated at 65°C for 15 min, followed by phenol-chloroform–isoamyl acetate (25:24:1) extraction and ethanol precipitation at −20°C overnight. MmeI-digested gDNA and an Illumina barcoded adaptor with two random base pair overhangs were ligated using T4 DNA ligase (Epicentre) for 1 h at 25°C. The transposon with 20 bp of genomic DNA sequence junction and ligated adaptor was amplified using Phusion High-Fidelity with GC buffer master mix (New England BioLabs) using primers (P1 M6 MmeI and Gex PCR Primer 2) that anneal to the inverted repeat of the transposon and the ligated Illumina adaptor. The PCR was terminated during linear amplification (26). The 120-bp product was gel purified and saved at −20°C. After Sanger sequencing verification using the primer Gex PCR Primer 2 for the presence of the unique barcode, the PCR product was sequenced using the Illumina HiSeq 2500 Rapid single-read 50-bp program. Three samples with unique barcodes were mixed in a single Illumina lane, generating ∼30 M quality-passing reads per sample. All sequencing was performed by the University of Minnesota Genomics Center. The primers and barcoded adaptors used in the construction of the Tn-Seq library are referenced by van Opijnen and Camilli (68).

### Mapping of transposon insertions.

For each Tn-Seq library, raw sequences were demultiplexed and extracted according to the unique barcode. The barcode and transposon sequences were trimmed, keeping only genome sequences that were at least 16 bp in length. Reads were then aligned to our G. sulfurreducens reference genome resequenced using a combination of short- and long-read assembly for this work (66) using Bowtie (version 1.1.2) (69), with no mismatches allowed and discarding reads that could map in more than one location. In a typical experiment, more than 90% of the reads were mapped to unique sites. After subtracting TA insertion sites in genes found to cause lethal fitness defects under our conditions, the G. sulfurreducens genome contains about 55,000 sites where the mariner transposon could insert and produce viable mutants. As we also discounted insertions in the first 5% of a feature, as these often do not produce a knockout phenotype (28), libraries recovered insertions in 64% of the available genomic TA sites that could produce viable mutants, providing an average of nearly 10 independent mutants per gene feature.

Approximately 2.7% of the insertional reads mapped to more than one location in the G. sulfurreducens genome due to redundancies. Of interest to our analysis, the *omcB* (GSU2739 to GSU2737) and *omcC* (GSU2735 to GSU2731) gene clusters are tandemly duplicated in the genome. Two genes in each cluster are 99 to 100% identical (GSU2739 and GSU2738, and GSU2733 and GSU2732) at the nucleotide level, preventing unique reads from mapping to these genes, while the 12-heme cytochrome genes *omcB* (GSU2737) and *omcC* (GSU2731) share 87% DNA sequence identity and still contained a few characteristic sites, allowing us to measure their fitness during growth. When the 20-bp MmeI-generated genomic DNA reads mapped ambiguously to more than one site, they were excluded from analysis, and genes containing such sites were flagged to account for possible low insertion density in the gene that would wrongly code it as essential. Similar issues limited the amount of data for the small triheme cytochromes encoded by *ppcA*, *ppcB*, *ppcC*, *ppcD*, and *ppcE* because there are few unique 20-bp regions in these small paralogs that are adjacent to TA sites. However, most ambiguous reads mapped to the 35 transposable elements and duplicated rRNA genes. All ambiguous mappings were excluded from the essentiality analysis but were included for total read normalization between barcoded libraries.

To estimate the severity of a phenotype, the effective change in doubling times for Tn-Seq mutants was calculated. The exponential-growth equation was applied using the parent library doubling time of 10 h for the duration of electrode growth, comparing the reads in a gene under electrode and fumarate conditions, where *x* is the apparent electrode growth doubling time (in hours). According to the conditions of the experiment, a log_2_ ratio of −2 after 72 h implies a mutant with a doubling time of ∼15 h.

$$\frac{\text{Reads}\;(\text{electrode})}{\text{Reads}\;(\text{parent})}\;=\;\frac{e^{\frac{\text{ln}\;2}{x}\;\times \;72}}{e^{\frac{\text{ln}\;2}{10}\;\times \;72}}$$

All of the Tn-Seq library sequence manipulations were performed in a Galaxy server hosted on the Minnesota Supercomputing Institute at the University of Minnesota (70).

### Scarless gene deletion in G. sulfurreducens.

Roughly 1 kb flanking the targeted gene(s) of interest was cloned into the *sacB*-carrying pK18mobsacB plasmid. To prevent disruption of the flanking genes, about 30 bp of the target gene sequence coding for a small peptide was cloned within the flanking region. The *sacB* plasmid was transformed into E. coli conjugative donor strain S17-1 to conjugate into the G. sulfurreducens recipient. One milliliter of fully grown G. sulfurreducens acetate-fumarate culture was pelleted on top of 1 ml of S17-1 culture carrying the *sacB*-carrying plasmid, mixed on top of a 0.22-μm-pore-size filter resting on acetate-fumarate agar plates in an anaerobic chamber, and incubated for 4 h before streaking the mixture onto acetate-fumarate plates with 200 μg/ml kanamycin. This procedure selected G. sulfurreducens culture with pK18mobsacB integrated into either flanking region of the gene since the plasmid cannot replicate in G. sulfurreducens. A scarless gene deletion mutant was selected on acetate-fumarate plates containing 10% sucrose and confirmed using PCR with primers flanking the deletion site, and in cases of large deletions, primers targeting inner regions of the cluster (66). The primers used to clone the flanking regions into pK18mobsacB and flanking primers to confirm gene deletions are listed in Table S1 in the supplemental material.

### Bacterial two-hybrid assay.

To test potential protein-protein interactions between EsnA (GSU1704) and EnsB/EsnC (GSU2220/GSU2222), a LexA-based two bacterial two-hybrid assay was used (71). In this system, the soluble domain of EsnA fused with a variant LexA DNA binding domain (DBD) is coexpressed with EsnB or EsnC fused to a WT LexA DBD from a compatible vector in the E. coli SU202 strain; *lacZ* transcription is controlled by a hybrid *lexA* operator site where only WT variant LexA DBD heterodimers can bind. Lower β-galactosidase activity indicates stronger protein-protein interactions between LexA DBD fusions. Coexpression of EsnA fused to variant LexA DBD with pSR658 encoding only the WT LexA DBD was used as a negative control. The plasmids used are listed in Table 3. The primers used to amplify and construct EsnABC-LexA DBD fusion proteins expressing plasmids are listed in Table S1. The coding sequence for the transmembrane domain of EsnA was omitted from the construction of the LexA DBD fusion plasmids.

### Tn-Seq raw sequencing data.

All raw read data needed to recreate or reanalyze these experiments are available in NCBI BioProject PRJNA290373. Illumina sequence data trimmed to remove transposon sequences and barcode sequences, containing only genomic DNA (.fastq) from each condition, are deposited in NCBI Sequence Read Archive with the following accession numbers: SRX2199236 (fumarate outgrowth, used for determining essentiality), SRX2199234 (parent library, used as the reference to compare between fumarate and electrode conditions), and SRX2199233 (concatenated with fastq headers HJHKHADXX and HJF5FADXX for electrode outgrowth replicates). Mapping files (.bam) for the fumarate outgrowth data set used to determine essentiality are deposited as SRX2199235. The reference G. sulfurreducens genome used for all mapping was resequenced and deposited as SRX1101230. Instructions on how to view read mapping against our reference genome using IGV is available in File S7.

## Supplementary Material

Supplemental material
