# Supplementary material for: Genome Scale Mutational Analysis of Geobacter sulfurreducens Reveals Distinct Molecular Mechanisms for Respiration and Sensing of Poised Electrodes versus Fe(III) Oxides
Source: J Bacteriol. 2017 Sep 5;199(19):e00340-17. doi: 10.1128/JB.00340-17 (PMC5585712; doi:10.1128/JB.00340-17)
Supplement: Supplemental material [file supp_199_19_e00340-17__index.html]

Supplemental material 

# Genome Scale Mutational Analysis of Geobacter sulfurreducens Reveals Distinct Molecular Mechanisms for Respiration and Sensing of Poised Electrodes versus Fe(III) Oxides

## Supplemental material

- Supplemental file 1 -

  Table S1 (Primers)

  XLSX, 12K
- Supplemental file 2 -

  Table S2 (Raw data)

  XLSX, 1005K
- Supplemental file 3 -

  Table S3 (Essential genes predicted in this study versus the *in silico* model of Mahadevan et al.)

  XLSX, 52K
- Supplemental file 4 -

  Table S4 (Genes found to be essential in the acetate/fumarate outgrowth deep sequencing library)

  XLSX, 61K
- Supplemental file 5 -

  Table S5 (Reactions associated with central metabolism)

  XLSX, 13K
- Supplemental file 6 -

  Table S6 (Functional and sequence homologs in the *G. sulfurreducens* genome)

  XLSX, 30K
- Supplemental file 7 -

  File S7 (Supplemental text)

  PDF, 67K
