# Supplementary material for: Genome Scale Mutational Analysis of Geobacter sulfurreducens Reveals Distinct Molecular Mechanisms for Respiration and Sensing of Poised Electrodes versus Fe(III) Oxides
Source: J Bacteriol. 2017 Sep 5;199(19):e00340-17. doi: 10.1128/JB.00340-17 (PMC5585712; doi:10.1128/JB.00340-17)
Supplement: Supplemental material [file JB.00340-17_zjb999094518s7.pdf]

Viewing read mapping data

To view raw mapping data against the *Geobacter sulfurreducens* genome used for these experiments, you need the GenBank genome file and the SAM mapping file (.sam). These can be viewed in a genome viewer such as IGV. The data used for essentiality has ~40,000,000 reads mapped after acetate donor/fumarate acceptor outgrowth experiments used to make essentiality calls.

The reference genome and reads used in this study are in our NCBI BioProject (PRJNA290373) but the fully annotated GenBank sequence can be easily downloaded from the Bond Lab GitHub repository. The SAM mapping file is also deposited in the NCBI Short Read Archive.

Reference genome with annotated features, sRNAs, riboswitch GEMM motifs, etc.
Bond Lab GitHub link: Geobacter_sulfurreducens_MN1.gbk
Right click 'Download' and select 'Save file as…'
Make sure filename ends in ".gbk" – some browsers append ".txt".

Read mapping file
NCBI SRA link: SRX2199235
Navigate to the "Alignment" tab
Output run in – select "SAM" and click on "File" to save it to your computer
Compressed file: SRR4304967_GSUL_MN1.4.sam.gz size: 340 MB
Uncompressed file size: 3.56 GB

Read mapping Index file
Will be generated in IGV – see below.

Download a browser such as IGV Genome browser

http://software.broadinstitute.org/software/igv/download

Instructions

Step 1: Install IGV

Step 2: Download the gbk genome reference file from the repository and the SAM (.sam) from the NCBI SRA and unzip.

Step 3: Open IGV, go to "Genomes", and click on "Load Genome from File…"

Step 4: Navigate to the folder with all the sequence files, and click on Geobacter_sulfurreducens_MN1.gbk

Step 5: Goto "File" and click on "Load from File…" and navigate to the sequence fold and click on SRR4304967_GSUL_MN1.4.sam

Step 6: IGV will prompt you to create a SAM index file, click "Go" to create one. It should take a few minutes.

Now that the read mapping is loaded, zoom using the “+” button in the top right corner, and browse by moving the read mappings left and right or search using the GSU# locus tags.

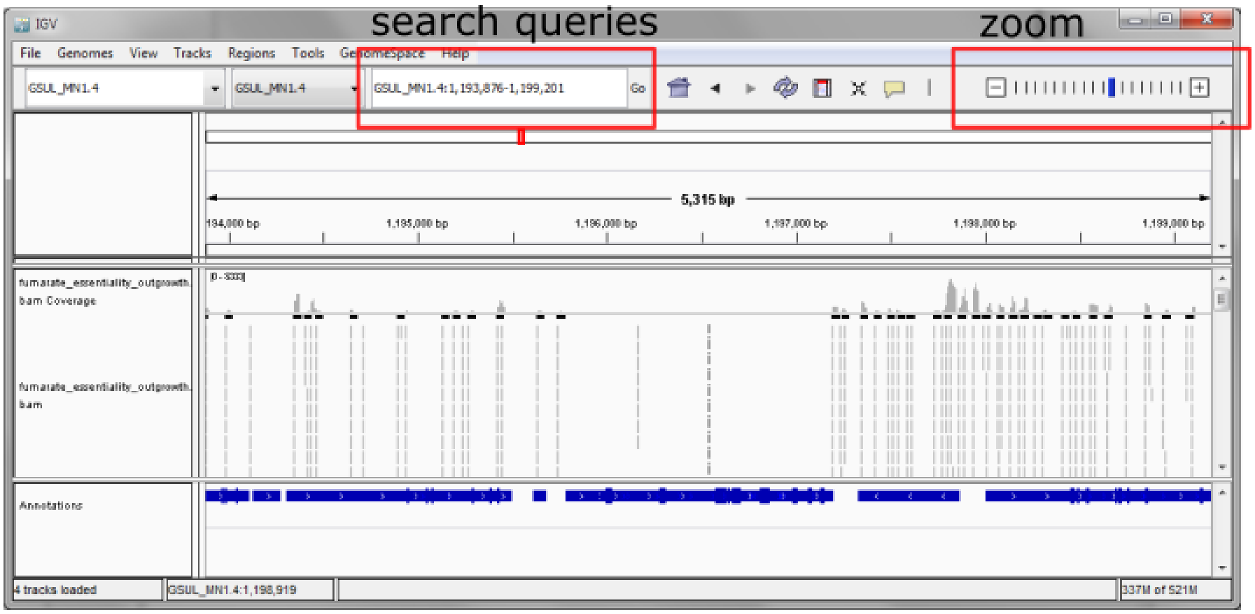

For example, search for either “frdB” or GSU1177 – a subunit of the essential fumarate reductase.
